# Efficient red light photo-uncaging of active molecules in water upon assembly into nanoparticles†
†Electronic supplementary information (ESI) available: Experimental procedures, additional chromatograms and spectra. See DOI: 10.1039/c5sc03717d


**DOI:** 10.1039/c5sc03717d

**Published:** 2015-12-23

**Authors:** Carl-Johan Carling, Jason Olejniczak, Alexandra Foucault-Collet, Guillaume Collet, Mathieu L. Viger, Viet Anh Nguyen Huu, Brendan M. Duggan, Adah Almutairi

**Affiliations:** a Skaggs School of Pharmacy and Pharmaceutical Sciences , University of California, San Diego , 9500 Gilman Dr. , La Jolla , California 92093 , USA . Email: aalmutairi@ucsd.edu; b Department of Chemistry and Biochemistry , University of California, San Diego , 9500 Gilman Dr. , La Jolla , California 92093 , USA; c Department of Nanoengineering , University of California, San Diego , 9500 Gilman Dr. , La Jolla , California 92093 , USA; d Department of Materials Science and Engineering , University of California, San Diego , 9500 Gilman Dr. , La Jolla , California 92093 , USA

## Abstract

We introduce a means of efficiently photo-uncaging active compounds from amino-1,4-benzoquinone in aqueous environments. Aqueous photochemistry of this photocage with one-photon red light is typically not efficient unless the photocaged molecules are allowed to assemble into nanoparticles. A variety of biologically active molecules were functionalized with the photocage and subsequently formulated into water-dispersible nanoparticles. Red light irradiation through various mammalian tissues achieved efficient photo-uncaging. Co-encapsulation of NIR fluorescent dyes and subsequent photomodulation provides a NIR fluorescent tool to assess both particle location and successful photorelease.

## Abstract 2

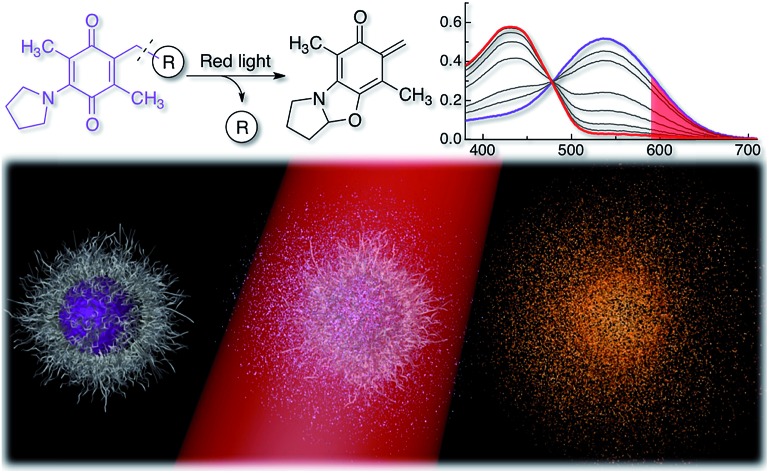
One-photon red visible light-responsive photocage–drug conjugate nanoparticles dissolve and release free drug upon irradiation.

## Introduction

External and non-invasive control over the chemistry and availability of active molecules, or the structural integrity of materials in aqueous environments has great potential to improve healthcare, aid scientific research and for applications in industry and agriculture.^1^–^6^ To achieve such control, light-responsive molecules are widely sought after, as light can be applied with high 2D and 3D spatial and temporal precision. Light driven chemistry for biological applications motivates the development of systems capable of functioning in aqueous environments, at higher efficiencies and ever-deeper light penetration into bulk turbid media such as mammalian tissues. 3D spatial resolution is of utmost importance to certain biological research applications.^7^–^12^ Recently research efforts from our group and others have developed several NIR laser activated chemistries *via* the absorption of two photons of NIR light.^13^–^23^ This allows for 3D spatial control compared with the 2D control allowed by the single photon process. Although the non-linear nature of the two-photon process yields the highly desired 3D spatial control, the process is not as efficient as single photon photochemistry, especially as scattering at deeper distances will necessitate refocusing of the laser with advanced laser technologies.^24^,^25^ Applications that require rapid bulk photochemistry in turbid media without 3D laser control would enjoy the benefits of higher efficiencies offered by the single photon process. Low power red light (600–700 nm) produced by cheap lamps is a promising candidate to activate long-wavelength absorbing photocages and photoswitches deep in bulk turbid media. The light has enough energy for efficient one-photon processes, mitigating the use of expensive high-power NIR laser sources, and can still innocuously penetrate mammalian tissues due to less absorbance. Single photon photochemistries such as release and photoswitching using low power red light have been reported,^26^–^37^ and research efforts toward this goal is a burgeoning research area.^6^,^38^–^47^

**Fig. 1:**
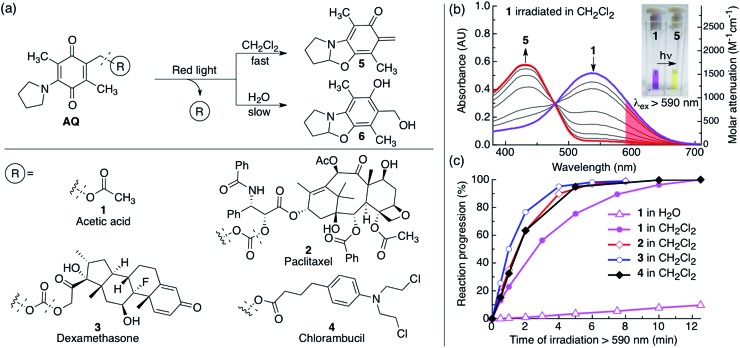
(a) (Top) Scheme illustrating the photorelease reaction of the AQ photocage upon irradiation in CH_2_Cl_2_ or H_2_O with red light and (bottom) structures of conjugates **1–4**. Hashed lines indicate bonds that break upon irradiation. (b) Changes in absorption of **1** dissolved in CH_2_Cl_2_ (0.34 mM, 0.5 mL) upon red light irradiation (*λ*_ex_ > 590 nm, 183 mW, 65 mW cm^–2^); red light absorption is shaded in red. Inset: photograph of the color change before (purple) and after irradiation (yellow). (c) Kinetics of the photoreaction of **1** dissolved in H_2_O (5% CH_3_CN, 0.34 mM, 0.5 mL) and **1–4** dissolved in CH_2_Cl_2_ (0.34 mM, 0.5 mL). Reaction progression = (1 – *A*/*A*_0_) × 100%, where *A* = absorbance at 535 nm.

To expand the available toolbox, we employed the amino-1,4-benzoquinone photocage developed by Chen and Steinmetz^26^,^27^ for its efficient red light single photon chemistry to photocage paclitaxel, dexamethasone, and chlorambucil. We chose these biologically active molecules to showcase the versatility of our approach and because they have previously been photocaged using other chemistries.^15^,^23^,^48^–^53^ The AQ photocage, which has not been employed since its development, has one-photon visible light absorption from 400–700 nm and allows fast (20–115 ms)^26^,^27^ and clean photorelease, with excellent photochemical yield (100% at 100% conversion)^26^,^27^ and quantum yield (*Φ*: 0.07–0.1 in CH_2_Cl_2_).^26^,^27^ However, water both degrades the chromophore and substantially suppresses its photochemical efficiency (*Φ*: 0.003–0.007 in 30% aq. CH_3_CN).^26^,^27^ The decreased aqueous photochemical efficiency is illustrated in Fig. 1c where compound **1** is irradiated in water (open triangles) and in CH_2_Cl_2_ (solid circles).

To overcome this water incompatibility, we formulated the hydrophobic photocage–drug conjugate molecules **2–4** into water-dispersible nanoparticles **P-2**, **P-3** and **P-4**, respectively. The corresponding particles' hydrophobic core protects the sensitive AQ chromophore from water so that the photochemistry functions efficiently and AQ resists degradation. Upon irradiation, the photocage–drug conjugate is efficiently photocleaved to yield the more hydrophilic free pristine drug, resulting in disassembly and release.

Nanoparticle formulation of the photocage–drug conjugate molecules eliminates the need for any toxic solubilizing excipients like Kolliphor EL^48^,^54^ or DMSO. Furthermore, formulation of photocage–drug conjugate nanoparticles provides a high loading and offers the opportunity to co-encapsulate additional cargo such as monitoring agents and additional drugs. Co-loading with NIR fluorescent molecules can provide valuable real-time information about particle location^23^ and release.^55^

We have introduced a means of efficiently photo-uncaging a variety of biologically active compounds from amino-1,4-benzoquinone in aqueous environments. To achieve photouncaging in aqueous environment the photocaged molecules were formulated into water dispersible nanoparticles with a hydrophobic core to circumvent the poor aqueous photochemistry of this photocage. Red light irradiation through various mammalian tissues achieved efficient photo-uncaging, demonstrating the practical potential of this system. Co-encapsulation of NIR fluorescent dyes and subsequent photomodulation provides a NIR fluorescent tool to assess particle location and successful photorelease.

## Results and discussion

### Photochemistry of conjugates **1–4**

We synthesized compounds **1–4** from a previously reported precursor^26^,^27^ (Fig. 1, Scheme S1†). We chose to functionalize the drugs paclitaxel (**2**), dexamethasone (**3**) and chlorambucil (**4**) with the AQ photocage, as these drugs are of clinical importance. Paclitaxel and dexamethasone were attached to AQ *via* a carbonate bond and chlorambucil was attached *via* an ester bond. We also synthesized the model compound **1**, where AQ is functionalized with acetic acid *via* an ester bond.

**Fig. 2:**
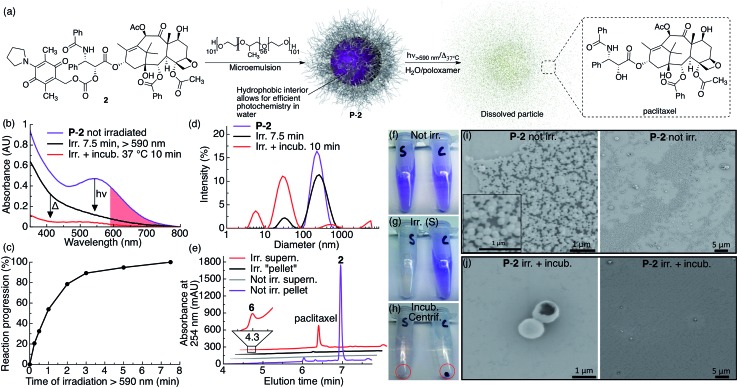
**P-2** photocage–drug conjugate nanoparticles dissolve and release drug upon red light irradiation. (a) Scheme illustrating the general preparation, composition and photochemistry of the **P-2** nanoparticles. (b) UV-vis absorption spectra of **P-2** (**2**: 0.1 mM, 1 mL) in water containing 1% w/v poloxamer 407 before irradiation (purple trace), after 7.5 min irradiation (black trace, *λ*_ex_ > 590 nm, 183 mW, 65 mW cm^–2^) and after 10 min incubation at 37 °C (red trace). The red shaded area highlights the red light region of absorption for **P-2**. (c) Kinetics of the same **P-2** sample upon irradiation with red light. (d) **P-2** particle size as measured by DLS before irradiation (purple trace), after 7.5 min irradiation (black trace) and after 10 min incubation at 37 °C (red trace). (e) HPLC chromatogram of separated and silica gel filtered (EtOAc) pellet and supernatant of irradiated and non-irradiated control samples. (f–h) Photographs of **P-2** samples (S: irradiated, C: not irradiated) (f) before irradiation, (g) after 7.5 min irradiation (tube S), and (h) after incubation (10 min) and centrifugation (the red circle highlight the centrifuged pellet). (i–j) SEM images of centrifugation-washed **P-2** pellets (i) without irradiation (inset in (i) is a magnification) and (j) after 7.5 min irradiation and 10 min incubation.

**Fig. 3:**
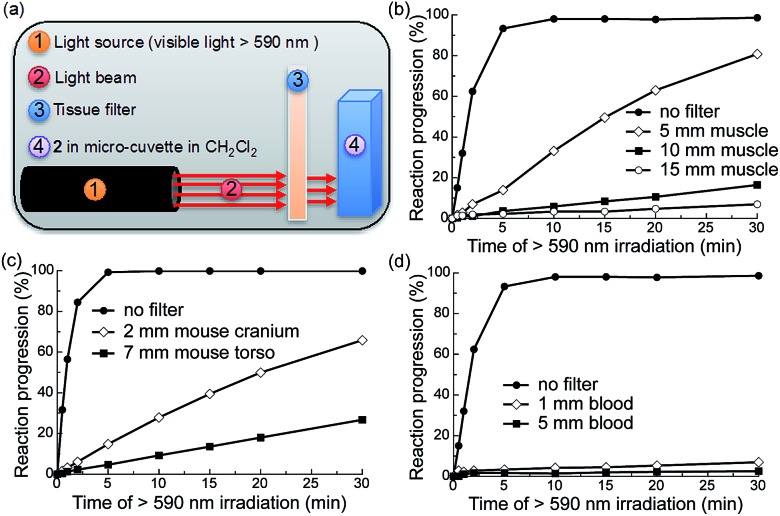
Photochemistry of **2** is possible upon irradiation through mammalian tissue filters containing muscle and bone. (a) Illustration of the experimental setup. (b–d) Photochemical kinetics of **2** in CH_2_Cl_2_ (0.34 mM, 0.5 mL) over increasing periods of irradiation with red visible light (*λ*_ex_ > 590 nm, 183 mW, 65 mW cm^–2^) with or without tissue filters in the beam path: (b) bovine muscle sandwiched between glass slides; (c) mouse cranium with skin or mouse torso with skin, ribs and muscle; (d) rat blood in cuvette. Reaction progression = (1 – *A*/*A*_0_) × 100%, where *A* = absorbance at 535 nm.

We studied the photoreaction of **1–4** by UV-vis spectroscopy, ^1^H NMR spectroscopy and high-pressure liquid chromatography with mass detection (HPLC-MS). We found that the photoreaction of **1–4** proceeded efficiently upon red light irradiation in CH_2_Cl_2_ (*λ*_ex_ > 590 nm, 183 mW, 65 mW cm^–2^) (Fig. 1 and S1†). The products of the photoreactions of **1–4** in CH_2_Cl_2_ or CDCl_3_ were identified as the ortho-quinone methide **5** (ref. 26 and 27) and the pristine drug (**1**: acetic acid, **2**: paclitaxel, **3**: dexamethasone and **4**: chlorambucil) (Fig. 1–3, S1 and S2†). **2–4** had similar kinetics when irradiated with red light in CH_2_Cl_2_ with a half-life of 1.2 ± 0.1 min, while **1** had a half-life of 2.6 min (Fig. 1c). In water the photochemical kinetics of water-soluble **1** were severely depressed, in line with previous studies^26^,^27^ (Fig. 1c). When we studied the photoreaction of **1** in H_2_O (4% CH_3_CN) the sample became colorless upon irradiation, and the water-captured compound **6** was formed instead of **5**, consistent with previous reports^25^,^26^ (Fig. 1a).

**Fig. 4:**
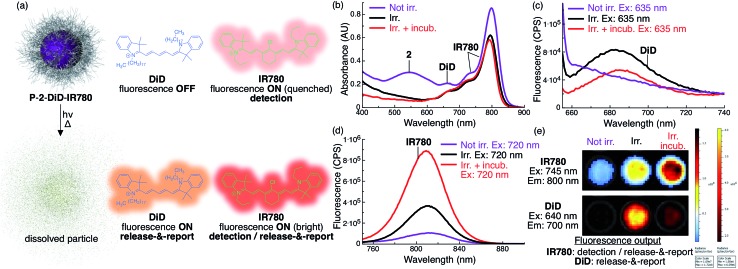
Increase in fluorescence of DiD and IR780 upon light-triggered release from **P-2** particles indicates particle status. (a) Scheme describing the fluorescent state of DiD and IR780 encapsulated in **P-2-DiD-IR780** particles before (top) and after irradiation with red light (bottom). (b) Absorbance of **P-2-DiD-IR780** particles (**2**: 9 × 10^–5^ M, 1 mL, DiD: 1 × 10^–6^ M (1 mol% loading compared to **2**), IR780: 5 × 10^–6^ M (6 mol% loading compared to **2**), in H_2_O with 1% w/v poloxamer 407) before (purple trace), after 7.5 min irradiation (*λ*_ex_ > 590 nm, 183 mW, 65 mW cm^–2^, black trace) and after irradiation and 10 min incubation at 37 °C (red trace). (c–d) Fluorescence of the same samples excited at (c) 635 nm (DiD channel) and (d) 720 nm (IR780 channel). (e) IVIS image of a well plate containing a similar **P-2-DiD-IR780** dispersion before irradiation (left well), after irradiation (middle well) and after irradiation and incubation (right well). Top, IR780 fluorescence channel (*λ*_em_ = 800 nm); bottom, DiD fluorescence channel (*λ*_em_ = 700 nm). Both rows are the same three wells.

Upon photolysis of the AQ photocage the absorption blue shifts, a highly beneficial property as it prevents the photoproducts from acting as inner filters or continuing to react upon prolonged irradiation.^23^,^56^ It is also advantageous as it allows easy gauging of the reaction progression by visual inspection and by UV-vis spectroscopy (Fig. 1b, 2a and g and S1†) and the change in absorption can be used to modulate fluorescence and thus indicate reaction progression (Fig. 4).

### Formulation and photochemistry of photocage–drug particles

Monodisperse, water-dispersible photocage–drug nanoparticles were formulated from the hydrophobic molecules **2–4** by micro-emulsion probe-sonication using poloxamer 407 (1% w/v) as surfactant to provide PEG coating of the particles (Fig. 2, S4 and S5, see ESI for experimental details†). The size of the particles was determined by SEM and DLS, and their composition was determined by ^1^H NMR spectroscopy by first drying the particles and then dissolving the material in CDCl_3_ (Table 1).

**Table 1:** Composition and size of photocage–drug conjugate nanoparticles **P-2**, **P-3** and **P-4**

Particle	Particle composition^*a*^		Particle size	
	Conjugate	Poloxamer	SEM	DLS
**P-2**	17 wt% 69 mol%	82 wt% 31 mol%	141 ± 10 nm	210 ± 10 nm PDI: 0.02
**P-3**	35 wt% 91.5 mol%	65 wt% 8.5 mol%	108 ± 20 nm	177 ± 64 nm PDI: 0.1
**P-4**	39 wt% 94 mol%	61 wt% 6 mol%	291 ± 58 nm	305 ± 101 nm PDI: 0.1

^*a*^Particles were dried, dissolved in CDCl_3_ and measured by ^1^H NMR spectroscopy (cryoprobe, 600 mHz).

We studied the photochemical behavior of **P-2**, **P-3** and **P-4** particles dispersed in aqueous solutions by UV-vis spectroscopy, DLS, SEM and HPLC-MS, and found that the photoreaction of AQ inside all the particles was very efficient and comparable to when the corresponding molecule is irradiated in CH_2_Cl_2_, thereby overcoming the challenge of efficiently activating AQ photochemistry in water (Fig. 1b and 2c). When **P-2** particles are irradiated in water containing poloxamer 407 (1% w/v), they dissolve upon brief irradiation with red light at room-temperature (7.5 min) followed by brief incubation at 37 °C (10 min) (Fig. 2).

**P-2** particle dissolution following irradiation and incubation can be clearly seen by the absence of a pellet after centrifugation (Fig. 2h, tube marked S) and the absence of small particles in SEM images (Fig. 2j). We further measured the dissolution of **P-2** by DLS, which showed a particle size decrease (Fig. 2d). We also observed a substantial decrease in scattering in the UV-vis spectrum, further indicating particle dissolution (Fig. 2b). Separating the centrifuged pellet from the supernatant and analyzing the silica gel filtered samples by HPLC-MS confirms that irradiated particles effectively release free paclitaxel into solution, whereas non-irradiated particles stay intact (Fig. 2e). As we only observed paclitaxel in the supernatant of the irradiated sample, we conclude that paclitaxel is efficiently released from **P-2** under these conditions. We noted that the **P-2** sample became colorless upon irradiation under these conditions, which is consistent with water-trapping of **5** and formation of **6** (Fig. 1, 2b, e and g).

We further studied the thermal stability of the **P-2** particles dispersed in water, water containing poloxamer 407 (1% w/v) and in fetal bovine serum (FBS, 10% and 100% v/v) incubated at 37 °C. We found that the particles were relatively stable over several hours (Fig. S3†). However, upon longer incubation times (days) we observed that the particles dispersed in water containing poloxamer 407 (1% w/v) and in 100% FBS dissolved (Fig. S3†). During these conditions we observed that the dissolved AQ chromophore slowly degraded and lost its purple color. It is thus interesting to note that the AQ chromophore is shielded from degradation in particle form whereas dissolved in aqueous media it degrades.

We studied the physical and photochemical properties of **P-3** and **P-4**. We found that both **3** and **4** could be readily formulated into nanoparticles (Table 1). While SEM images of **P-3** showed spherical particles, **P-4** particles looked like pancakes (Fig. S5†). This is due to flattening of the **P-4** particles on the SEM grid upon sample preparation as **4** is an oil at room temperature, while both **2** and **3** are solids. Nevertheless, we found that that the kinetics of the **P-3** and **P-4** particles' photochemistry when dispersed in water was comparable to irradiation in CH_2_Cl_2_. We further found that both **P-3** and **P-4** released their corresponding drug more efficiently than **P-2** upon irradiation at room temperature (Fig. S4 and S5†); this was expected because of the greater hydrophilicity of dexamethasone and chlorambucil compared to paclitaxel. Upon HPLC-MS analysis of the separated and silica gel filtered pellet and supernatant, we observed the corresponding drug mainly in the supernatant of the irradiated samples, indicating efficient release (Fig. S5d–g†).

Hydrophobic AQ-drug conjugates allow formulation of nanoparticles, providing a suitable environment for AQ photochemistry to function effectively in water. This approach thus expands the photo caging chemistry of AQ in aqueous environments to efficiently release drugs in water using low-power red light.

### Irradiation through mammalian tissue filters

To determine the efficiency of this system upon scattering and absorption of light through bulk turbid media we investigated the extent to which various mammalian tissues attenuate the ability of red light to trigger AQ photochemistry. To this end, we irradiated compound **2** through a number of tissue filters with varying scattering and absorption profiles (Fig. 3a): 5, 10, and 15 mm muscle (bovine) sandwiched between glass slides (Fig. 3b); hairless mouse cranium with skin and bone (2 mm) (Fig. 3c); hairless mouse torso with skin, ribs and muscle (4–7 mm) (Fig. 3c); and 1 and 5 mm-thick samples of rat blood (Fig. 4d). We measured the photochemical kinetics of **2** in CH_2_Cl_2_ by UV-vis absorption spectroscopy, monitoring disappearance of absorption at 535 nm (where complete disappearance was considered 100% reaction progression). We chose to irradiate **2** in CH_2_Cl_2_ to ensure detailed kinetic analysis by avoiding detrimental scattering by the nanoparticles dispersed in water.

We observed that the photoreaction of **2** proceeded quite efficiently when irradiated through bovine muscle filters, where reaction progression reached 83%, 16%, and 5% after 30 min irradiation through 5, 10, and 15 mm muscle tissue, respectively (Fig. 3b). When irradiated through the mouse cranium and torso filters, reaction progression reached 66% and 22% after 30 min of irradiation, respectively (Fig. 3c). However, due to the strong absorption of heme, 5 mm blood allowed only ∼2% photocleavage, whereas 1 mm blood allowed 7% after 30 min irradiation. This indicates that photorelease would likely be inefficient in larger blood vessels such as arteries or veins (Fig. 3d). Based on these results we thus conclude that the photochemistry of **2** can be triggered relatively efficiently at depths up to ∼0.5–1 cm in mammals. As photocaged derivatives **3** and **4** possess similar photolytic efficiencies, they should behave similarly upon irradiation through tissue filters.

### Loading **P-2** with DiD and IR780 provides fluorescent indication of particle location and release in real time

To produce an NIR fluorescent system that can be used to locate particles and assess photorelease, we co-loaded **P-2** particles with the fluorescent cyanine dyes DiD and IR780 (**P-2-DiD-IR780**, Fig. 4, see ESI for experimental details†).

Since the absorbance of DiD overlaps with both the AQ chromophore and IR780, the fluorescence of DiD is fully quenched inside the intact non-irradiated particles (Fig. 4a and c). Upon irradiation, **2** is photocleaved into molecules whose absorbance does not overlap with DiD, resulting in activation of fluorescence, reporting the photocleavage reaction (Fig. 4a–c and e). However, we observed that upon incubation and full dissolution of the particles, the DiD signal decreased in intensity (Fig. 4c and e). This phenomenon is due to a lower fluorescence efficiency of DiD in water.

Towards locating intact particles, we co-loaded IR780. Since this long-wavelength absorbing dye's fluorescence only minimally overlaps with the absorption of AQ and DiD, it fluoresces inside intact non-irradiated particles, albeit with a dim quenched signal (Fig. 4a, b, d and e). Upon irradiation and particle dissolution, IR780 fluorescence is substantially enhanced (9×) due to loss of absorbance overlap with **2** and DiD, leading to a second release signal (Fig. 4a, b, d and e).

Because of the significant differences in excitation/emission wavelengths and fluorescence intensity between the two fluorophores depending on their environment, particle location and release can be measured by multichannel NIR fluorescence imaging.

## Conclusions

By utilizing the amino-1,4-benzoquinone (AQ) photocage, we synthesized photocage–drug conjugate nanoparticles responsive to tissue-penetrating one-photon red light. Upon brief irradiation with low-power red light, the particles dissolve and release the pristine drug in aqueous media. Particle formulation provides both high drug loading and a water-shielded environment, allowing efficient photocleavage and minimizing water degradation of the AQ chromophore. Light-induced NIR fluorescence modulation of co-encapsulated IR780 and DiD provides a non-invasive means of detecting particles' location and release. This work highlights the practical potential of one-photon red light-responsive systems for non-invasive light-triggered release in bulk turbid media such as mammalian tissues.

## Supplementary Material

Supplementary information
