# Supplementary material for: Efficient red light photo-uncaging of active molecules in water upon assembly into nanoparticles
Source: Chem Sci. 2015 Dec 23;7(3):2392–8. doi: 10.1039/c5sc03717d (PMC4800316; doi:10.1039/c5sc03717d)
Supplement: Supplementary file 1 [file SC-007-C5SC03717D-s001.pdf]

Electronic Supplementary Material (ESI) for Chemical Science.
This journal is © The Royal Society of Chemistry 2015

Supporting Information

Efficient Red Light Photo-Uncaging of Active Molecules in Water Upon Assembly into Nanoparticles

Carl-Johan Carling[a], Jason Olejniczak[b], Alexandra Foucault-Collet[a], Guillaume Collet[a], Mathieu L. Viger[a], Viet Anh Nguyen Huu[c], Brendan M. Duggan[a] and Adah Almutairi [a,c,d]*

[a]Skaggs School of Pharmacy and Pharmaceutical Sciences, [b]Department of Chemistry and Biochemistry, [c]Department of Nanoengineering, [d]Department of Materials Science and Engineering. University of California, San Diego, 9500 Gilman Dr., La Jolla, California 92093, USA.

*Corresponding author: Adah Almutairi, UCSD, 9500 Gilman Dr., La Jolla, California 92093, USA. Phone: (858) 246-0871, E-mail: aalmutairi@ucsd.edu

General methods and instrumentation

All chemicals and solvents were purchased from Sigma-Aldrich and used as received unless specified. Compound **7** was synthesized according to a published procedure.[1] Silica gel flash column chromatography was performed using an automated CombiFlash® Rf 200 system. ^{1}H NMR and ^{13}C NMR spectra were acquired using a Varian spectrometer working at 600 MHz and 150 MHz respectively. Chemical shifts (δ) are reported in ppm relative to TMS, and coupling constants (*J*) are reported in hertz. High-resolution mass spectra were acquired using an Agilent 6230 ESI-TOFMS in positive ion mode. Low-resolution HPLC-MS chromatograms were acquired on an Agilent Technologies 1260/1290 Infinity HPLC-MS equipped with a ZORBAX 1.8 µm (2.1x50 mm) SB-C18 column and 6120 Quadrapole detector and run in a gradient of water and acetonitrile with 0.1% formic acid. UV-visible absorption spectra were collected using a Shimadzu UV-3600 UV-Vis spectrophotometer. Fluorescence spectra were acquired using a Horiba Jobin Yvon spectrofluorimeter. Particles were imaged using an Agilent 8500FE scanning electron microscope. Particle size was measured using a Malvern Zetasizer Nano-ZS. All samples were irradiated with long-wavelength visible light using a EXR 300W 82V halogen projector lamp in a Kodak Ektagraphic III AM slide projector filtered through a cut-on 590 nm color glass filter (Newport FSQ-OG590). Light power was measured using a Newport 1936-R optical power meter. Fluorescence imaging was performed using an IVIS Spectrum Pre-Clinical In Vivo Imaging System (Perkin-Elmer).

Experimental procedures

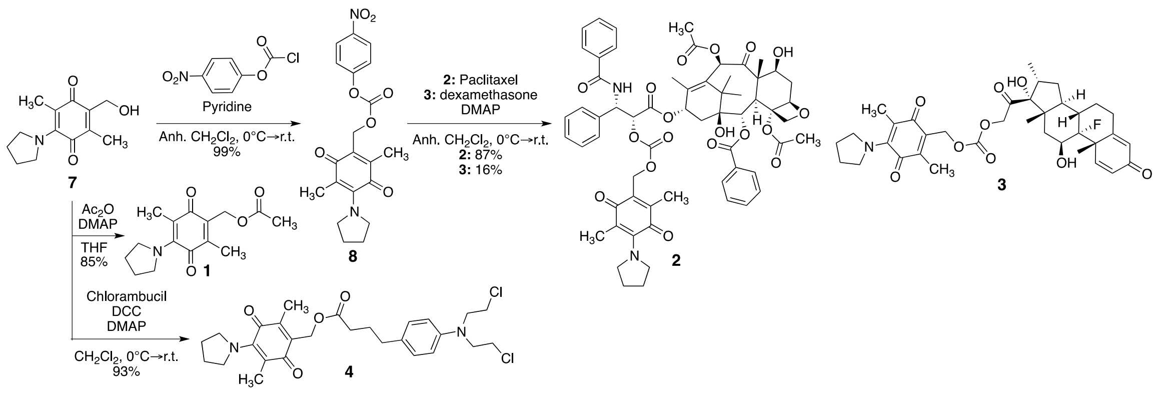

Scheme S1. Synthesis of compounds **1**-**4** and **8**. Compound **7** was synthesized according to a published procedure.[1]

Synthesis of 1

A solution of **7**[1] (43 mg, 0.18 mmol, 1 eq.) and DMAP (34 mg, 0.28 mol, 1.5 eq.) in THF (2 mL) was cooled to 0 °C. Acetic anhydride (0.026 mL, 0.28 mmol, 1.5 eq.) was added dropwise. The reaction was allowed to reach ambient temperature and stirred for 1.5 h, at which point the solvent was evaporated. The residue was purified by silica column chromatography (gradient hexanes → 10% EtOAc in hexanes) to yield the title compound **1** as a purple oil (43 mg, 0.155 mmol, 85% yield, stored at -20 °C wrapped in foil).

^{1}H NMR (600 MHz, $CDCl_3$) δ 5.02 (2H, s), 3.65-3.63 (4H, m), 2.05 (3H, s), 2.04 (6H, s), 1.88-1.86 (4H, m)

^{13}C NMR (150 MHz, $CDCl_3$) δ 187.50, 184.23, 170.78, 150.43, 141.20, 137.16, 57.97, 53.15, 25.99, 20.94, 13.19, 12.13

HRMS (ESI-TOFMS) calc. mass $(C_{15}H_{20}NO_4)^+$ $[M+H]^+$ 278.1387 Da, experimental mass $[M+H]^+$ 278.1384 Da, delta ppm: -1.1

Synthesis of 8

An oven dried round bottom flask and stir bar was cooled to ambient temperature under high vacuum before being backfilled with argon. The flask was charged with **6**[1]

(320 mg, 1.36 mg, 1 eq.) and p-Nitrophenyl chloroformate (548 mg, 2.72 mmol, 2 eq.). The system was evacuated and backfilled with argon. Anhydrous CH_2Cl_2 (5 ml) was added via syringe and the resulting solution was cooled to 0 °C. Anhydrous pyridine (0.22 mL, 2.72 mmol, 2 eq.) was added dropwise via syringe and the reaction was subsequently removed from the cold bath and wrapped in foil. After 30 min the reaction mixture was loaded onto a 24g Silica column and eluted with a gradient 100% hexanes → 10% → 30% EtOAc in hexanes under low-light conditions. The title compound **8** was isolated as a dark purple, highly viscous and sticky oil, which foams under high vacuum. (540 mg, 1.35 mmol, 99% yield, stored at -20 °C wrapped in foil).

^{1}H NMR (600 MHz, $CDCl_3$) δ 8.27 (2H, d, *J* = 9.18 Hz), 7.39 (2H, d, *J* = 9.18 Hz), 5.25 (2H, s), 3.68-3.66 (4H, m), 2.13 (3H, s), 2.10 (3H, s), 1.92-1.91 (4H, m)

^{13}C NMR (150 MHz, $CDCl_3$) δ 186.86, 183.27, 155.36, 152.03, 150.19, 145.12, 141.68, 135.09, 125.07, 121.59, 111.64, 62.17, 53.01, 25.69, 12.89, 12.07

HRMS (ESI-TOFMS) calc. mass $(C_{20}H_{21}N_2O_7)^+$ $[M+H]^+$ 401.1343 Da, experimental mass $[M+H]^+$ 401.1340 Da, delta ppm: -0.7

Synthesis of 2

An oven dried round bottom flask and stir bar was cooled to ambient temperature under high vacuum before being backfilled with argon. The flask was charged with **8** (48.7 mg, 0.12 mg, 1.25 eq., vial was first frozen in N_2 to make **8** solid and non-sticky) and DMAP (29.7 mg, 0.24 mmol, 2 eq.) and then evacuated and backfilled with argon twice. Anhydrous CH_2Cl_2 (1 mL) was added via syringe and the resulting solution was cooled to 0 °C. The flask was opened briefly and paclitaxel (83.1 mg, 0.097 mmol, 1 eq., Fisher) was added. The system was briefly evacuated and backfilled with argon twice. The reaction was removed from the cold bath and wrapped in foil. After 30 min the reaction mixture was loaded onto a 12 g Silica column and eluted with a gradient 100% hexanes → 40% EtOAc in hexanes under low-light conditions. The title compound **2** was isolated as a dark purple solid. (94 mg, 0.084 mmol, 87% yield, stored at -20 °C wrapped in foil).

^{1}H NMR (600 MHz, $CDCl_3$) δ 8.15 (2H, d, *J* = 7.3 Hz), 7.77 (2H, d, *J* = 7.4 Hz), 7.60 (1H, t, *J* =7.4 Hz), 7.53-7.47 (3H, m), 7.40-7.38 (6H, m), 7.34-7.31 (1H, m), 7.07 (1H, d, *J* = 9.3 Hz), 6.29-6.26 (2H, m), 6.00 (1H, dd, *J* = 9.3, 2.3 Hz), 5.69 (1H, d, *J* = 7.1 Hz), 5.46 (1H, d, *J* = 2.6 Hz), 5.15 (2H, q, *J* = 11.5 Hz), 4.97 (1H, d, *J* = 9.5 Hz), 4.45-4.12 (1H, m), 4.32 (1H, d, *J* = 8.5 Hz), 4.20 (1H, d, *J* = 8.5 Hz), 3.80 (1H, d, *J* = 7.1 Hz), 3.64 (4H, m), 2.58-2.53 (1H, m), 2.44 (3H, s), 2.42-2.38 (1H, m), 2.24 (3H, s), 2.21-2.17 (1H, m), 2.04 (1H, s), 2.02 (3H, s), 2.00 (3H, s), 1.92 (3H, s), 1.90 (1H, s), 1.89-1.87 (4H, m), 1.68 (3H, s), 1.24 (3H, s), 1.14 (3H, s)

^{13}C NMR: See Table S1 for shifts and assignments.

HRMS (ESI-TOFMS) calc. mass $(C_{61}H_{67}N_2O_{18})^+$ $[M+H]^+$ 1115.4383 Da, experimental mass $[M+H]^+$ 1115.4379 Da, delta ppm: -0.4

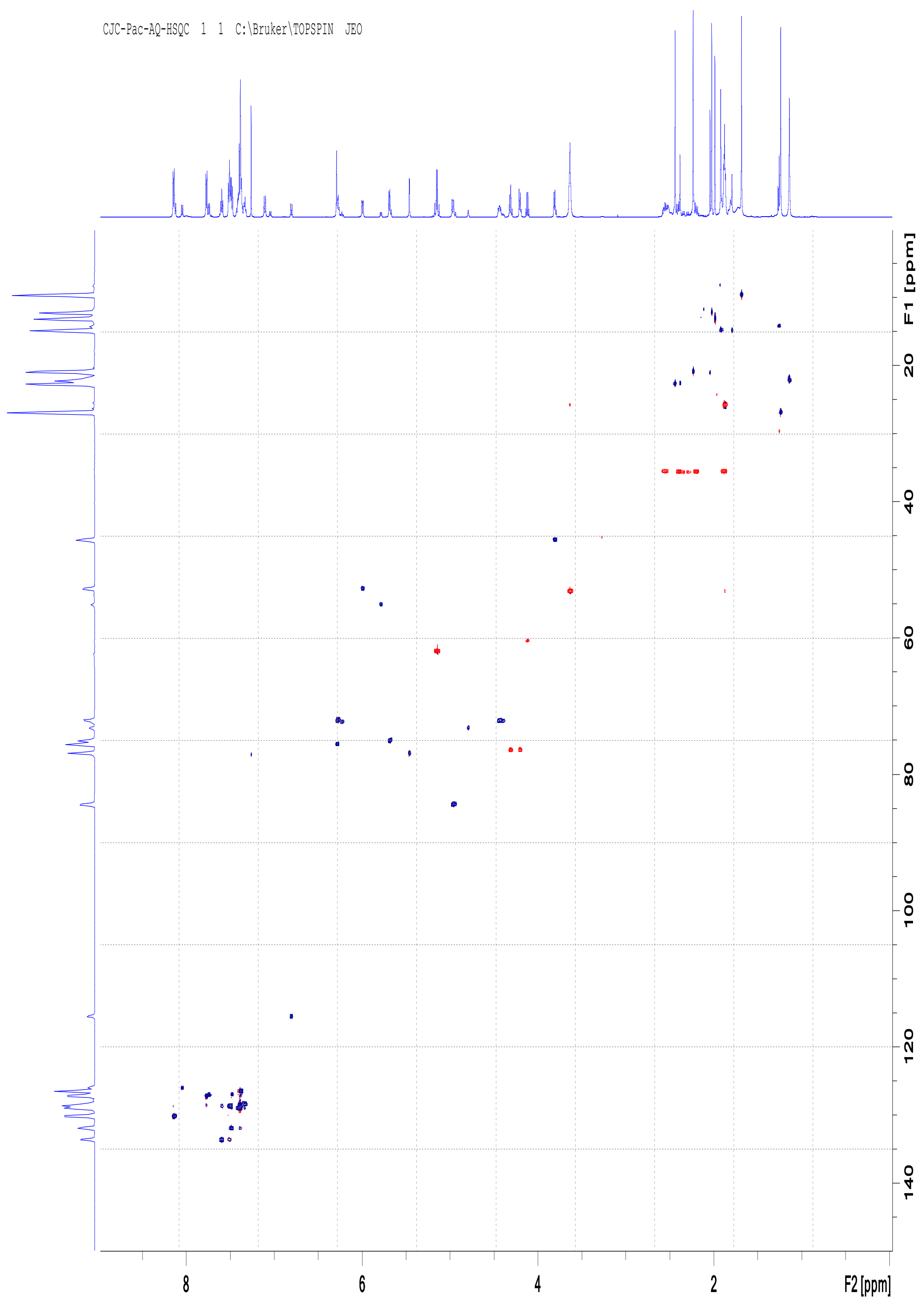
CJC-Pac-AQ-HSQC 1 1 C:\Bruker\TOPSPIN JEO
F1 [ppm]
20
40
60
80
100
120
140
8
6
4
2
F2 [ppm]

HSQC of **2**

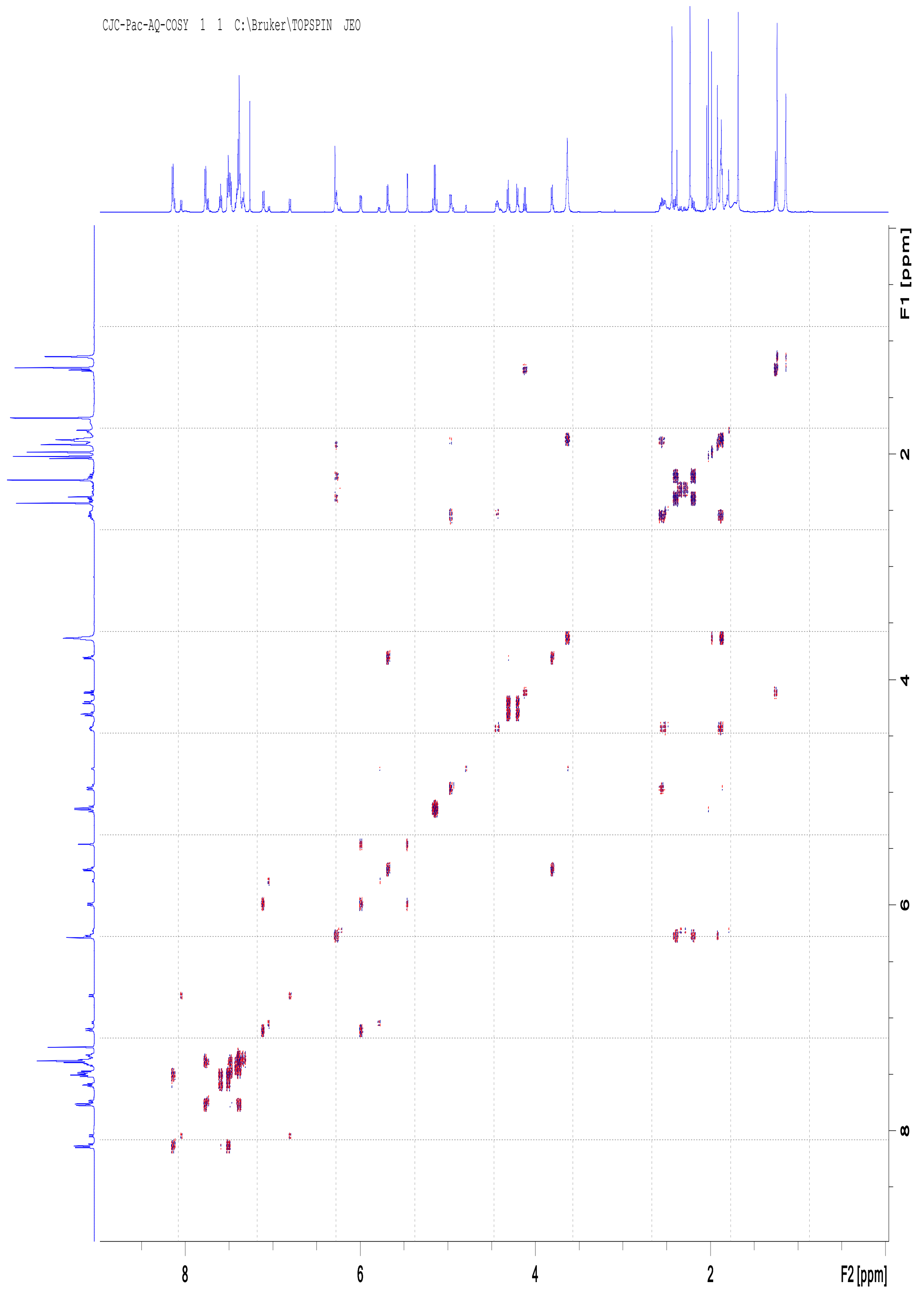

COSY of **2**

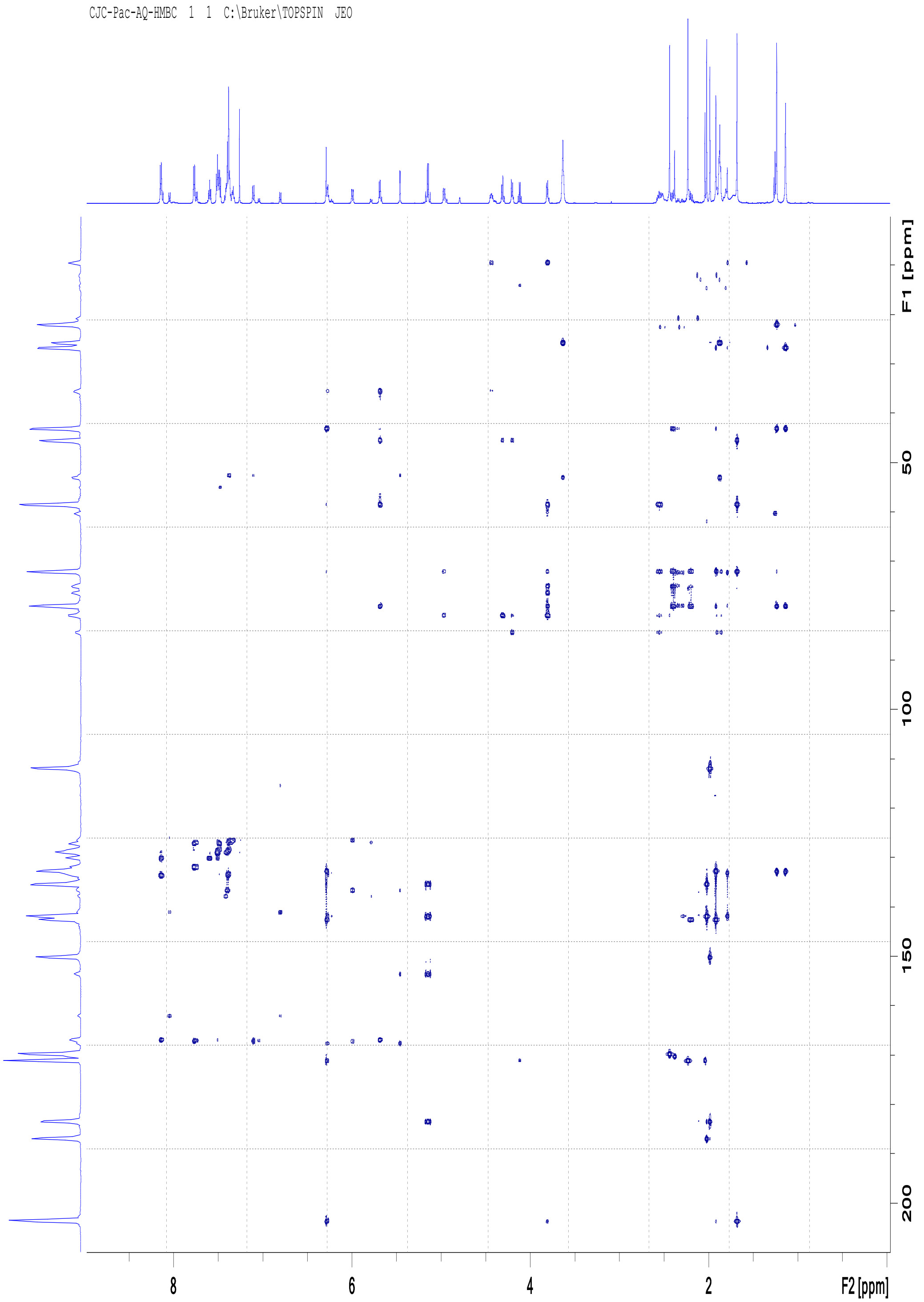
CJC-Pac-AQ-HMBC 1 1 C:\Bruker\TOPSPIN JEO
F1 [ppm]
50
100
150
200
8
6
4
2
F2 [ppm]

HMBC of **2**

Position	Proton Shift(s) (ppm)	Carbon Shifts (ppm)
1	2.44	22.8
2		170.0
3		81.2
4	4.31, 4,20	76.5
5	6.27	72.0
6	2.56,2.20	35.9
7	4.42	72.1
8		58.7
9	1.68	9.6
10	3.80	45.9
11	5.69	75.6
12		43.4
13	1.14	22.2
14	1.24	27.0
15		132.9
16	4.44	72.2
17		204.0
18		171.4
19	2.24	22.3
20		142.9
21	1.92	14.6
22	6.27	75.9
23	2.40,2.20	35.6
24		79.3
25		167.2
26		133.6
27	8.15	130.2
28	7.51	128.9
29	7.60	133.8
30		167.3
31	5.46	77.0
32	6.00	52.7
33		167.9
34		131.9
35	7.77	127.4
36	7.38	128.6
37	7.48	132.0
38		136.9
39	7.38	126.3
40	7.39	129.3
41	7.33	128.4
42		153.8
43	5.15	62.1
44		135.7
45		183.7
46		112.1
47	2.00	13.0

48		150.5
49	3.64	53.4
50	1.88	26.0
51		187.2
52		142.2
53	2.02	12.6

Table S1. Assignments of ^{1}H and ^{13}C resonances based upon 2d NMR spectral data for **2**.

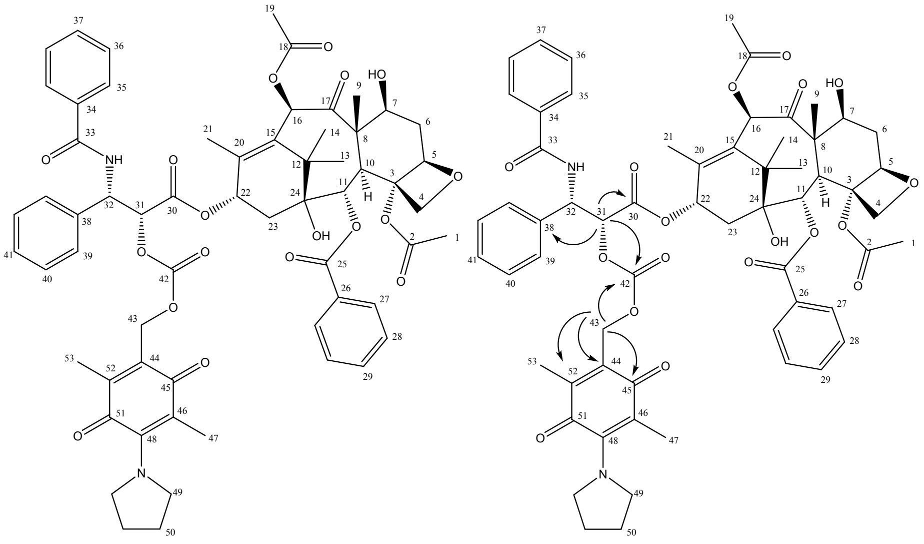

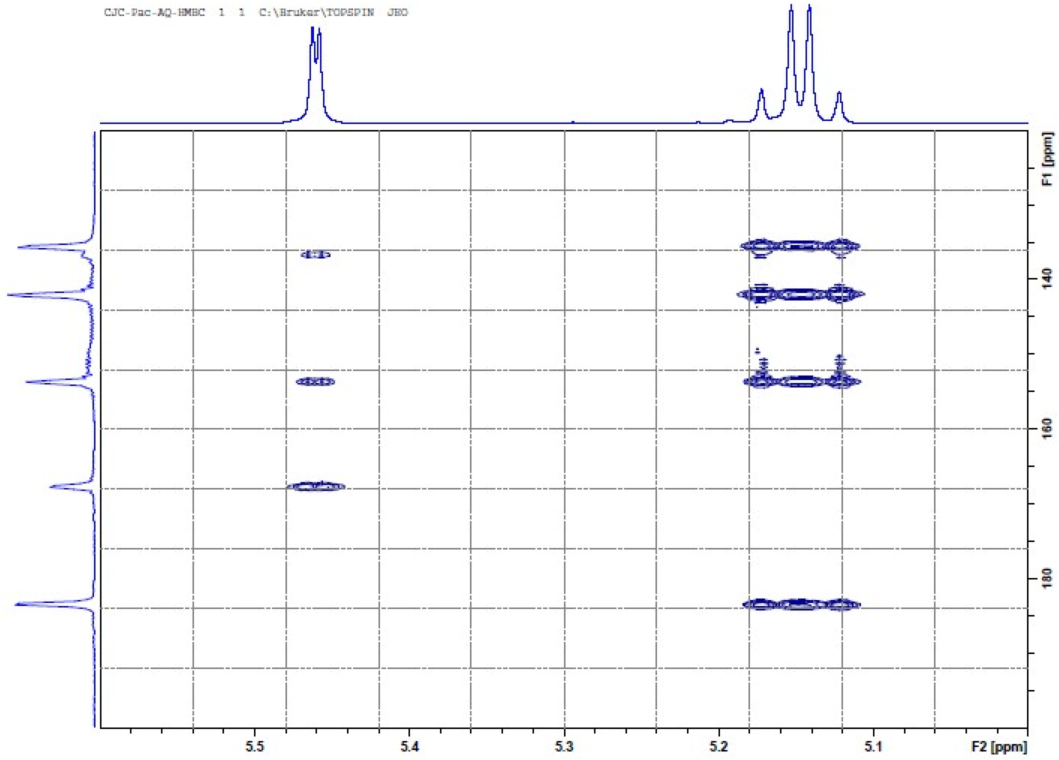

Critical region of **2** HMBC expanded. In it you can see correlations between the protons

of position 43 and the carbons of 42, 44, 45, and 52, also the correlations between proton of 31 and carbons of 30, 38 and 42. This confirms the location of the AQ protecting group.

Synthesis of 3

An oven dried round bottom flask and stir bar was wrapped in foil and cooled to ambient temperature under high vacuum before being backfilled with argon. The flask was charged with **8** (101.5 mg, 0.25 mmol, 1 eq.) and dexamethasone (109.9 mg, 0.28 mmol, 1.1 eq.). The system was evacuated and backfilled with argon. Anhydrous DMF (0.1 ml) was added to dissolve dexamethasone and then anhydrous CH_2Cl_2 (5 mL) was added and the solution was cooled to 0 °C in an ice bath. The flask was briefly opened and DMAP (62.3 mg, 0.51 mmol, 2 eq.) was added. The flask was purged and removed from the cold bath. After 6 h the brownish (note: DMAP degrades AQ) reaction was poured into a separatory funnel and diluted with EtOAc and washed with 4x60 ml brine. The aqueous phase was extracted once and the combined organic phases were dried over $MgSO_4$, vacuum filtered and concentrated. The crude mixture was loaded onto a 40 g Silica column from CH_2Cl_2 and eluted with a gradient 100% hexanes → 40% EtOAc in hexanes under low-light conditions. The title compound **3** was isolated as a dark purple solid. (27 mg, 0.04 mmol, 16% yield, stored at -20 °C wrapped in foil).

^{1}H NMR (600 MHz, $CDCl_3$) δ 7.27 (1H, d, *J* = 11.8 Hz), 6.33 (1H, d, *J* = 11.8 Hz), 6.10 (1H, s), 5.15 (2H, q, *J* = 7.5, 11.4 Hz), 4.90 (2H, q, 17.8, 18.4 Hz), 4.36 (1H, d, *J* = 8.6 Hz), 3.65 (1H, d, *J* = 3.9 Hz), 3.13-3.08 (1H, m), 2.64-2.58 (1H, m), 2.47-2.29 (5H, m), 2.11 (4H, s), 2.01 (3H, s), 1.89-1.87 (4H, m), 1.82-1.80 (1H, m), 1.78-1.72 (1H, m), 1.65 (1H, d, *J* = 14.2 Hz), 1.56 (3H, s), 1.06 (3H, s), 0.90 (3H, d, *J* = 7.3 Hz)

^{13}C NMR: See Table S2 for shifts and assignments.

HRMS (ESI-TOFMS) calc. mass $(C_{36}H_{45}FNO_9)^+$ $[M+H]^+$ 654.3073 Da, experimental mass $[M+H]^+$ 654.3076 Da, delta ppm: 0.5

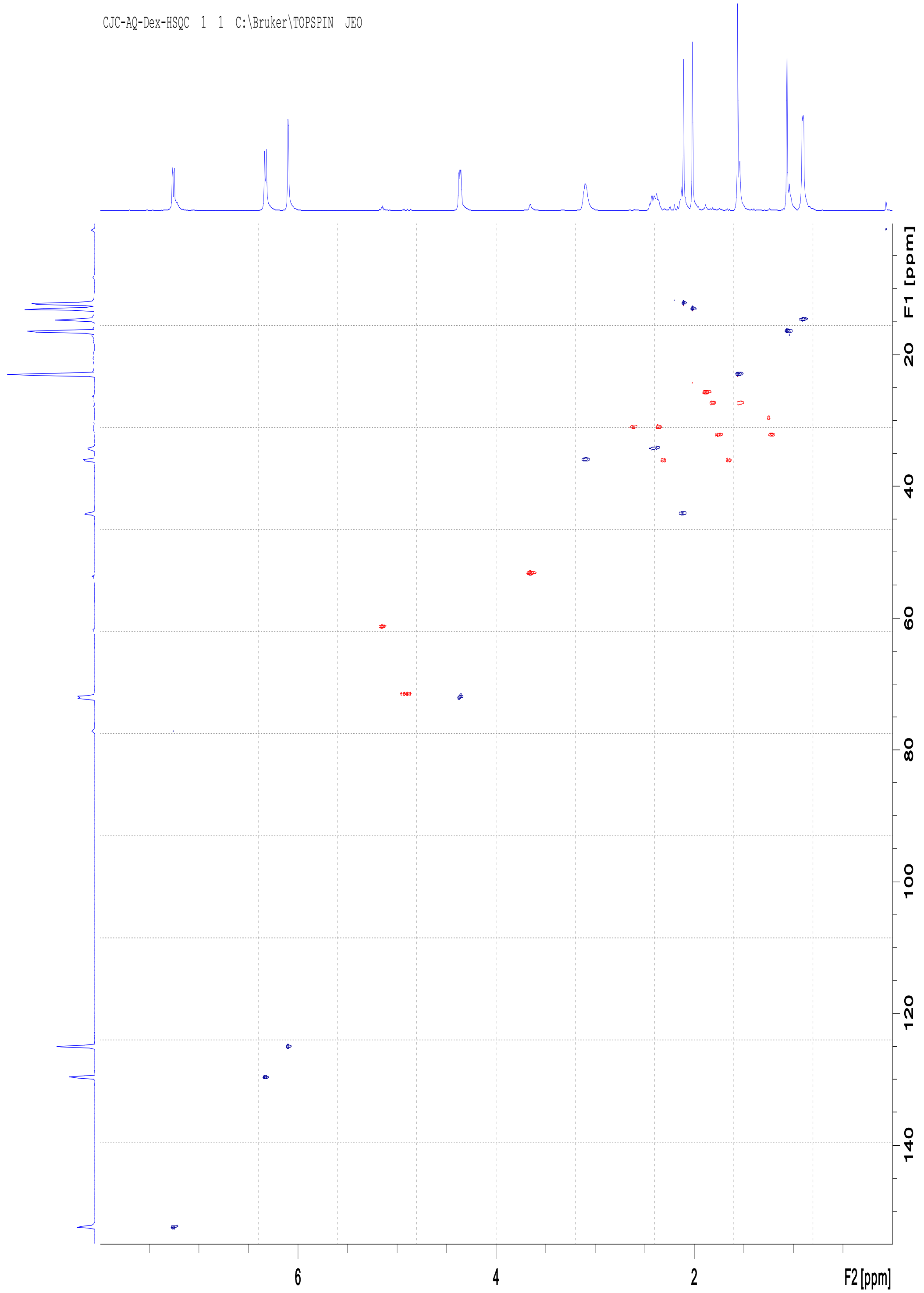

HSQC spectra of **3**

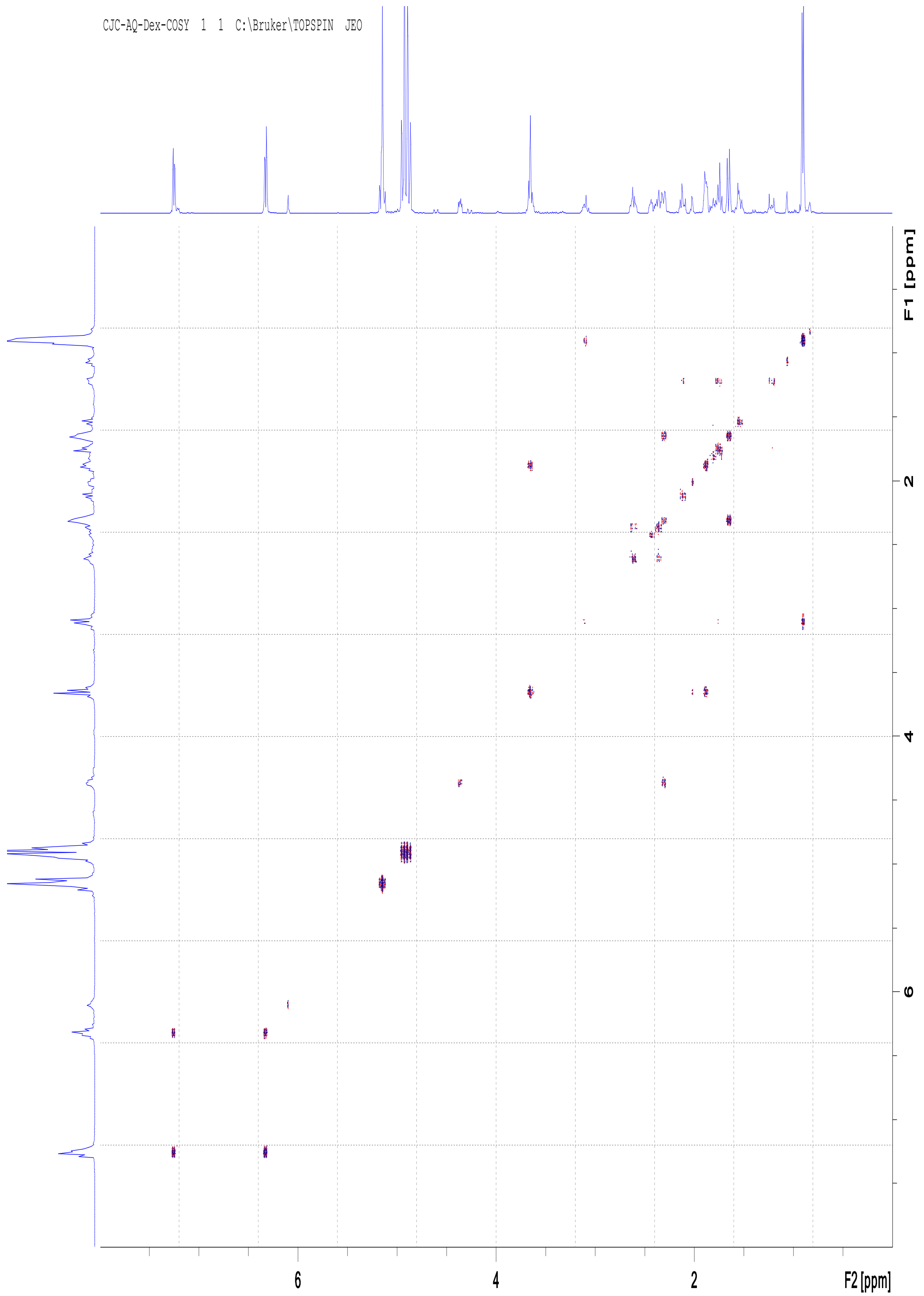

COSY Spectra of **3**

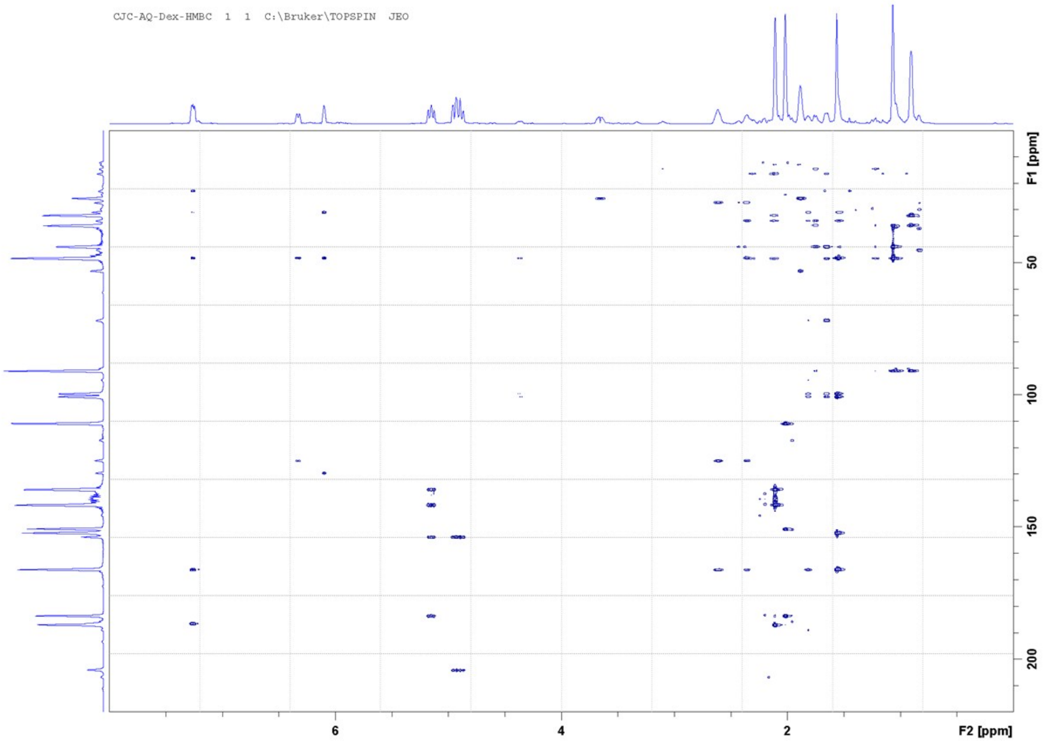

HMBC spectra of **3**

Position	Proton Shift(s) (ppm)	Carbon Shifts (ppm)
1	7.27	152.7
2	6.33	129.8
3		186.9
4	6.10	125.2
5		166.6
6	2.62,2.36	31.1
7	1.81,1.54	27.5
8	2.43	34.4
9	2.12	44.2
10	1.76,1.24	32.3
11	3.11	36.2
12	0.90	14.8
13		91.3
14		48.6
15	1.06	16.5
16	2.31,1.65	36.2
17	4.36	72.0
18		100.1
19		48.4
20	1.56	23.1
21		204.6
22	4.90	71.7

23		154.1
24	5.15	61.5
25		136.1
26		183.9
27		111.0
28	2.01	13.2
29		151.1
30	3.65	53.4
31	1.89	25.9
32		187.4
33		141.9
34	2.11	12.4

Table S2. Assignments of ^{1}H and ^{13}C resonances based upon 2d NMR spectral data for **3**.

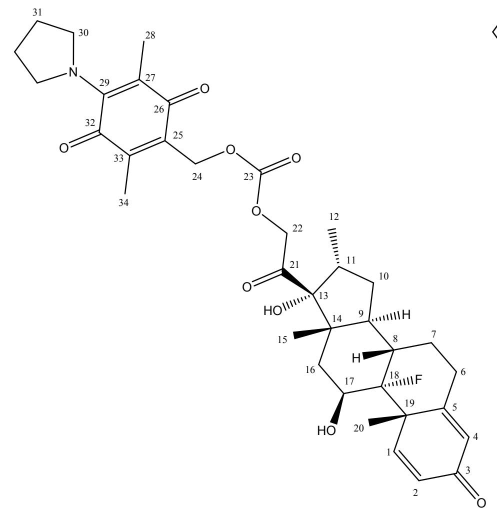

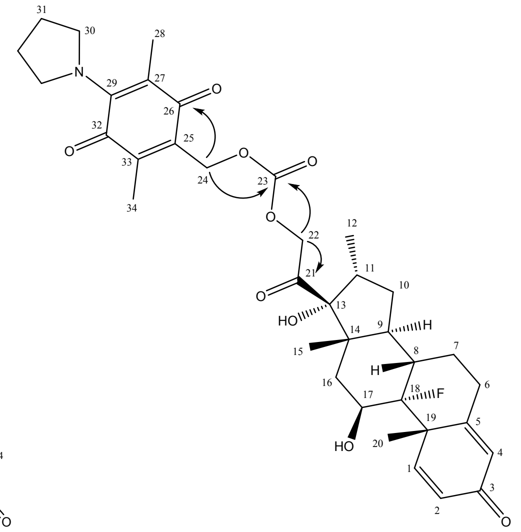

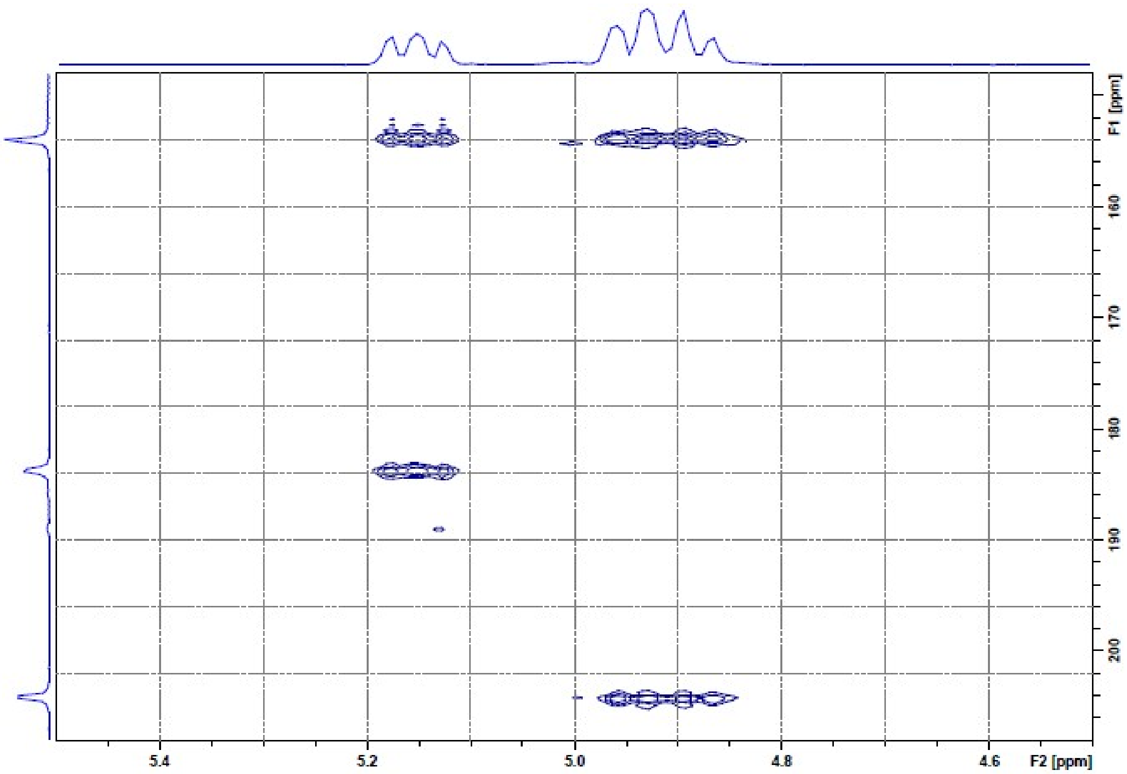

Critical region of **3** HMBC expanded. In it you can see correlations between the protons of position 22 and the carbons of 21 and 23, also the correlations between protons of 24 and carbons of 23 and 26. This confirms the location of the AQ protecting group.

Synthesis of 4

An oven dried round bottom flask and stir bar was wrapped in foil and cooled to ambient temperature under high vacuum before being backfilled with argon. The flask was charged with **7**[1] (99.6 mg, 0.42 mmol, 1 eq.), chlorambucil (191.7 mg, 0.63 mmol, 1.5 eq.) and DMAP (155.1 mg, 1.27 mmol, 3 eq.). The system was evacuated and backfilled with argon. Anhydrous CH_2Cl_2 (6 mL) was added and the solution was cooled to 0 °C in an ice bath. The flask was briefly opened and DCC (262 mg, 1.27 mmol, 3 eq.) was added. The flask was purged and removed from the cold bath. After 2 h under low-light conditions the reaction mixture was filtered through a silica plug and washed with

CH_2Cl_2. The crude mixture was concentrated and loaded onto a 12 g Silica column from CH_2Cl_2 and eluted with 20% EtOAc in hexanes under low-light conditions. The title compound **4** was isolated as a dark purple oil. (203 mg, 0.39 mmol, 93% yield, stored at -20 °C wrapped in foil).

^{1}H NMR (600 MHz, $CDCl_3$) δ 7.05 (2H, d, *J* = 8.5 Hz), 6.62 (2H, d, *J* = 8.5 Hz), 5.02 (2H, s), 3.70 (4H, t, *J* = 6.7 Hz), 3.65-3.60 (8H, m), 2.53 (2H, t, *J* = 7.6 Hz), 2,31 (2H, t, *J* = 7.6 Hz), 2.04 (3H, s), 2.03 (3H, s), 1.89-1.86 (6H, m)

^{13}C NMR (150 MHz, $CDCl_3$) δ 194.9, 187.5, 184.1, 173.4, 143.8, 141.1, 141.0, 129.9, 112.4, 90.5, 57.8, 53.8, 53.2, 40.6, 34.1, 33.5, 26.9, 26.0, 13.2, 12.2

HRMS (ESI-TOFMS) calc. mass $(C_{27}H_{34}N_2O_4Na)^+$ $[M+Na]^+$ 543.1788 Da, experimental mass $[M+Na]^+$ 543.1786 Da, delta ppm: -0.4

General procedure for particle formulation

In a 40 mL vial was added poloxamer 407 (0.1 g) and MilliQ-water (10 mL). The dispersion was carefully heated in a microwave oven. When the poloxamer 407 was dissolved, 6 mL was filtered through a 1 µm ceramic syringe filter into a new vial equipped with a stir bar. The solution was cooled to 0 °C in an ice bath. Conjugate **2**, **3** or **4** (10 mg) and any additives (IR780, 0.3 mg; DiD, 0.3 mg) was dissolved in CH_2Cl_2 (0.3 mL) and added to the water-poloxamer solution. While placed in the ice bath, the mixture was probe-sonicated (10 W, 8 min). At regular intervals the mixture was swirled. The vial was then wrapped in foil and put under low vacuum while stirring; the initial vacuum was adjusted with an air-intake needle equipped with an air-filter. When the dispersion stopped foaming under full vacuum, the air-inlet needle was removed and the particle dispersion was stirred for 1 hour in an ice bath. In the case of **P-4**, the mixture was sonicated once after the organic solvent was evaporated to produce a nice dispersion (50 W, 30 s). The dispersions were then put in the fridge over night. The dispersion was then transferred to a 35 mL Nalgene centrifuge tube, diluted with MilliQ water (14 mL) and centrifuged (21000 rpm, 20 min, 10 °C). The supernatant was removed using an electrical pipette, taking care not to disturb the pellet. The pellet was redispersed in

MilliQ water (20 mL) by sonication and the washing procedure was repeated twice more. The pellet was then redispersed in 2 mL MilliQ water and stored in an eppendorf tube wrapped in foil in the fridge. The concentration of the chromophores in the particle stock samples was measured by UV-Vis spectroscopy: 50 µL stock solution was dissolved in 950 µL CH_3CN and the absorbance was measured and compared to a concentration plot of the respective compounds. To elucidate the concentration/loading of DiD and IR780 in the **P-2-DiD-IR780** particle, the sample was irradiated to bleach the absorbance of **2**. The DiD and IR780 concentration was then approximated (due to partial absorbance overlap) against concentration plots of DiD and IR780. The composition of the particles was measured by ^1H NMR spectroscopy in $CDCl_3$.

HPLC analysis of the photoreaction of photocage-drug conjugate nanoparticles

Particles were dispersed in MilliQ water with 1% poloxamer 407. After irradiation the samples were centrifuged (21000 rpm, 20 min) and the supernatant and pellet were separated, concentrated, purified through a small silica plug (EtOAc eluent) to remove poloxamer 407 and dissolved in MeOH prior to analysis.

Tissue filters

The UCSD Institutional Animal Care and Use Committee approved the study.

Blood filter

Whole rat blood was placed in a cuvette with 5 mm or 1 mm depth. The cuvettes were then wrapped in foil so that light only could travel through the blood. The filter was taped to a cuvette holder and the setup was wrapped in foil to ensure only light filtered through the filter could reach the sample. The sample was placed inside the cuvette holder and irradiated. The changes in absorbance were monitored over time.

Muscle filter

Raw bovine muscle (5 mm thick beef from the grocery store) was cut into 4 cm by 2 cm rectangles and placed between two glass slides (1 slice = 5 mm, 2 slices = 10 mm and 3 slices = 15 mm). The corresponding assembly was then taped to a cardboard

with a cutout rectangular hole. The corresponding filter was then taped to a cuvette holder and the setup was wrapped in foil to ensure only light filtered through the filter could reach the sample. The sample was placed inside the cuvette holder and irradiated. The changes in absorbance were monitored over time.

Mouse filters

A mouse (BalbC) was shaved, euthanized and dissected. The brainless cranium (with skin) and the torso (with skin, ribs and muscle) were prepared. The corresponding mouse body part was taped to a cardboard with a cutout hole. The corresponding filter was then taped to a cuvette holder and the setup was wrapped in foil to ensure only light filtered through the filter could reach the sample. The sample was placed inside the cuvette holder and irradiated. The changes in absorbance were monitored over time.

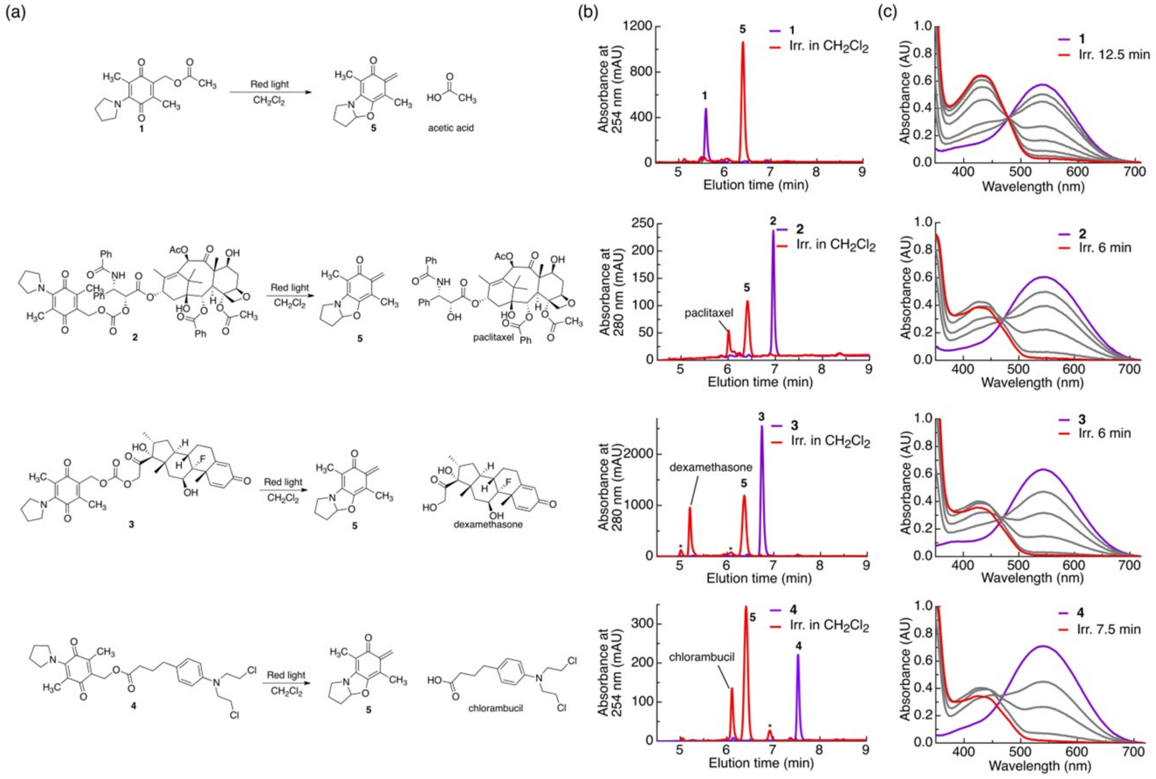

Figure S1. Photochemistry of conjugates **1**-**4**. (a) Schemes describing the photoreaction of each conjugate. (b) HPLC-MS analysis of the photoreaction. (c) Changes in the UV-Vis

spectra upon irradiation (λ_{ex} > 590 nm, 183 mW, 65 mWcm^{-2}). (*indicates unidentified compounds)

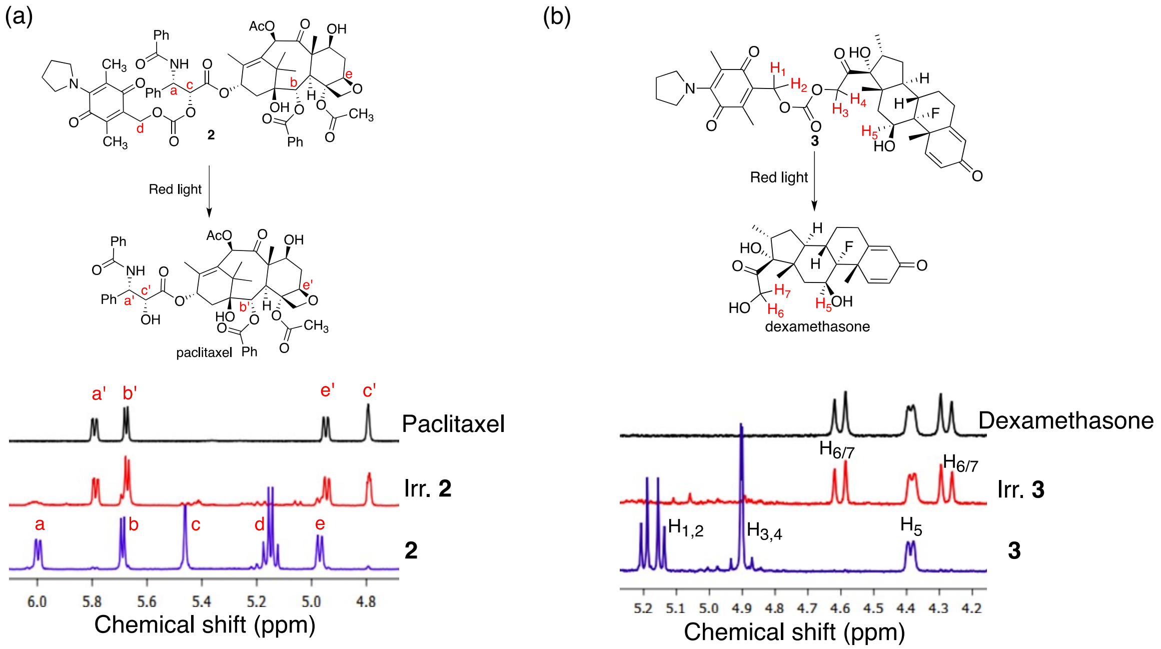

Figure S2. Photorelease yields the pristine drug. Top: photochemical reaction scheme and bottom: NMR data of (a) **2** and (b) **3** as they are irradiated in $CDCl_3$.

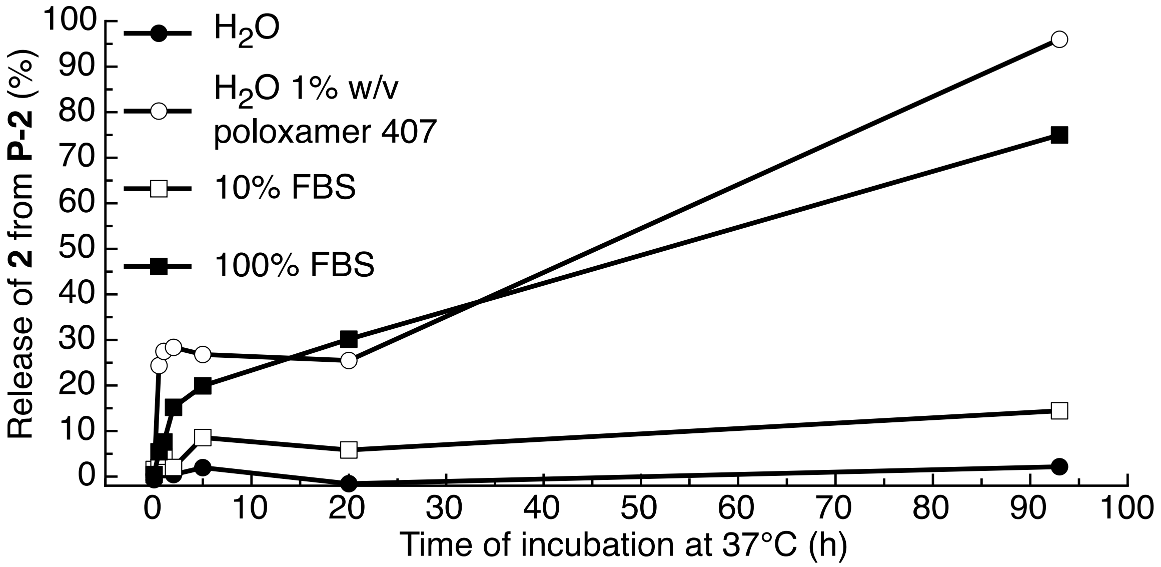

Figure S3. Stability of **P-2** in various solvents incubated at 37 °C. The amount of released **2** from **P-2** was measured by centrifuging the samples at regular intervals and measuring the absorbance at 550 nm of the supernatant. As **2** was found to slowly degrade when

dissolved, the last data point was obtained by comparing the absorbance of the redispersed pellet to the original value.

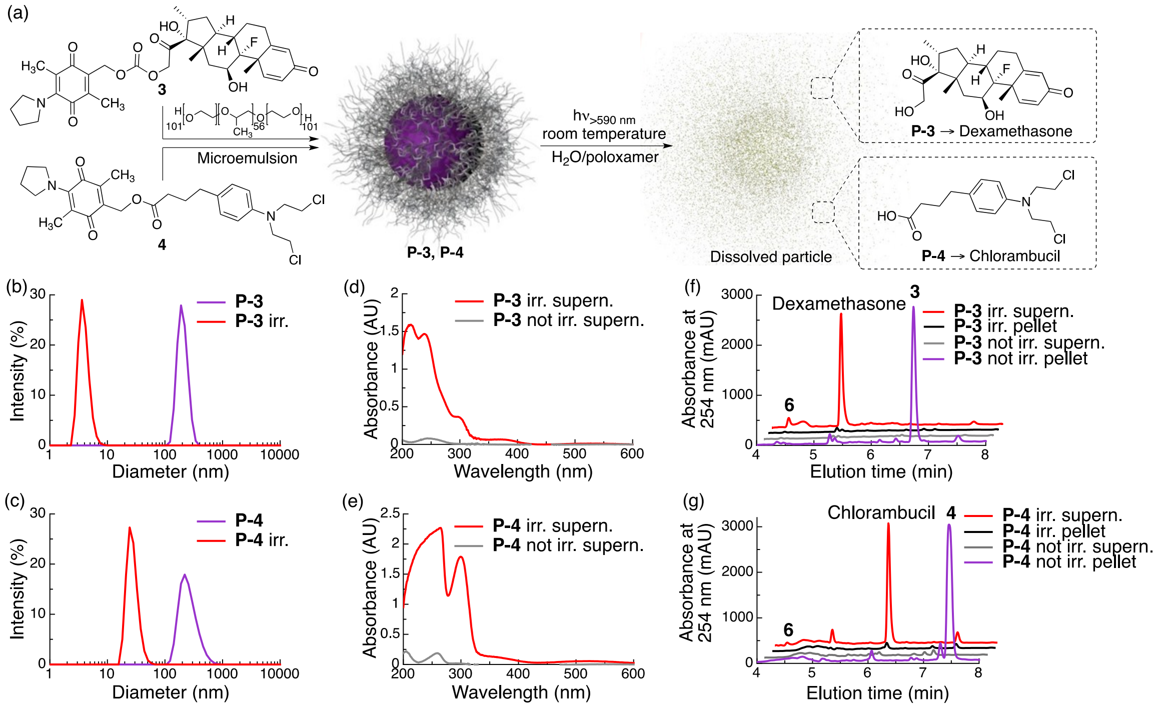

Figure S4. Irradiation of **P-3** and **P-4** with red light at room temperature releases the active drug. (a) Scheme illustrating the general preparation, composition and photochemistry of the **P-3** and **P-4** nanoparticles. (b-c) DLS traces of (b) **P-3** and (c) **P-4** particles before and after irradiation with red light (7.5 min, λ_{ex} > 590 nm, 183 mW, 65 mWcm^{-2}) at room temperature. (d-e) UV-vis absorption of the centrifugation separated supernatant of irradiated and non-irradiated samples of (d) **P-3** and (e) **P-4** particles. (f-g) HPLC-MS analysis of separated and silica gel filtered pellet and supernatant of irradiated and non-irradiated samples of (f) **P-3** and (g) **P-4** particles.

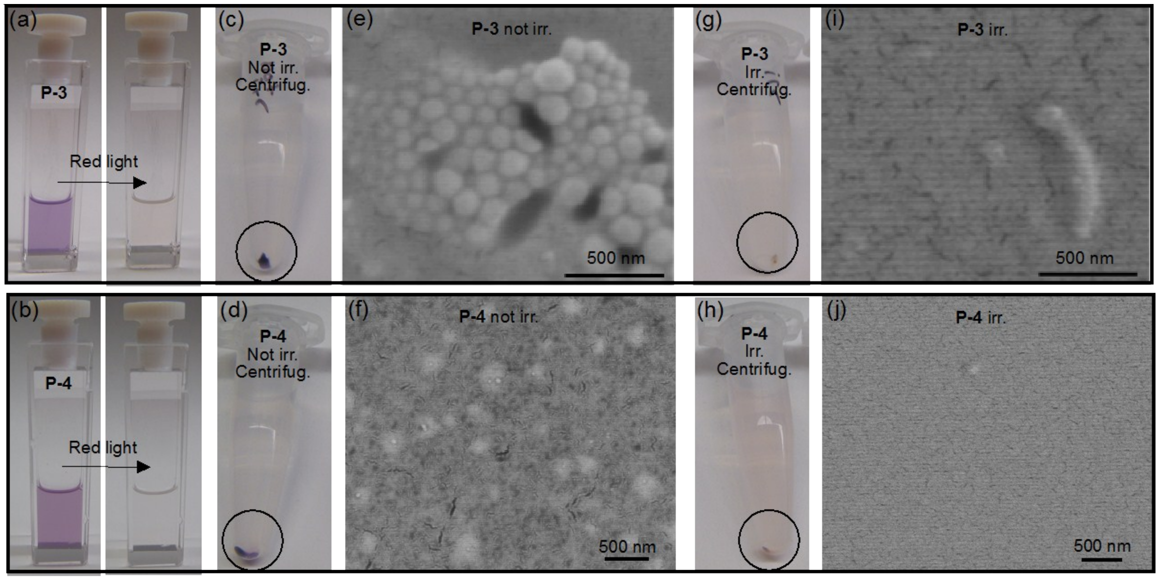

Figure S5. Photolysis of **P-3** and **P-4** particles. (a-b) Changes in color of (a) **P-3** and (b) **P-4** particles before and after irradiation. (c-d) Centrifuged pellet of non-irradiated (c) **P-3** and (d) **P-4** particles. (e-f) SEM images of the pellet of non-irradiated (e) **P-3** and (f) **P-4** particles. (g-h) Centrifuged pellet of irradiated (c) **P-3** and (d) **P-4** particles. (i-j) SEM images of the pellet of irradiated (e) **P-3** and (f) **P-4** particles.

References

1. (a) Chen, Y. G.; Steinmetz, M. G. *Org. Lett.* 2005, **7**, 3729. (b) Chen, Y. G.; Steinmetz, M. G. *J. Org. Chem.* 2006, **71**, 6053.
